# Proof of the Periodical Ripening and Separation of the Ova of Mammiferæ and Man, Independent of Coitus, Being the First Condition of Their Propagation

**Published:** 1845-04

**Authors:** 


"*Proof of the Periodical Ripening and Separation of the Ova of Mammalia and Man, independent of Coitus, being the first condition of their Propagation.* By Th. L. W. Bischoff, M. D., P. D., &c., Giessen, 1844."

In the March number of our last volume, we presented to our readers parts of a review of the above works. The extracts there made, showed forth the new theories of "Fecundation in the Mammiferæ," and the "Ripening and Separation of the Ova," with the proofs from experiments upon animals. It remains to show that these rules are true as regards the human family. We again take up the Review, and present entire the portion devoted to the consideration of this part of the subject.

"It is necessary, still, to prove the applicability of this law to woman, and to show that menstruation actually corresponds with the period of the maturation of the ova in their ovaries. Without resorting to the facts furnished by M. Négrier, (who, by the way, was the first to give an accurate account of some of the true anatomical characteristics of the discharge of ova by women, with a description of the successive evolutions of the Graafian vesicles, from the moment of their formation until the ova separate from the ovary,) M. Gendrin, Robert Lee, Paterson, T. Wharton Jones, &c., which are, in themselves, conclusive, we will merely adduce the proof afforded by the authors whose works we are considering.

"M. Pouchet, after accumulating a great number of proofs of the existence of a periodical menstrual discharge among animals, or, at any rate, when this is not observed, of a periodical afflux of blood into the genital organs, which is essentially connected with the rutting season, now known to correspond with the maturation of a Graafian vesicle, contends that the same phenomenon in women must be due to the same causes, or that menstruation in them must correspond with the period of maturation of an ovum.

"M. Raciborski, in addition to this mode of reasoning, describes the actual progressive development (pp. 53, 54, 92, 419,) of the Graafian vesicles in the ovaries of women, as he has done also of those in the sow, in proportion as we approach the age of puberty. 'In proportion as the ova ripen,' he says, 'the follicles which enclose them increase in size. Ten or twelve days before the *ponte*, they already become prominent above the surface of the ovary, sometimes in a nipple form, sometimes as a broad protuberance, having still semi-transparent parietes, and enclosing a yellowish-white viscid liquid, rich in granulations, visible by means of a microscope, and coagulating by means of alcohol, boiling water and nitric acid. At this period, if the body is opened shortly after death, the ovule may be quite easily distinguished in the midst of the granulations. The whole ovary becomes the seat of a strong congestion, and augments sensibly in volume.'—p. 421.

"'In proportion as the Graafian vesicles are developed, and the moment of *ponte* approaches, the parietes of the vesicle, although more and more distended, begin to appear less diaphanous, in consequence of the thickening of the internal membrane and of the hæmorrhage which occurs at the last moments in the interior of the vesicle. Finally, the point becomes evident at which the opening is to take place, at the most prominent part of the tumor,

This point generally presents the aspect of a reddish spot some lines in extent, caused by a strong injection, and even partly due to a slight effusion of blood into the thickness of the tunics of the vesicles.'—p. 424.

"This injection of the ovaries corresponds with a strong congestion of all the genital organs of the female, which finally relieves itself by a hæmorrhage, constituting the menstrual discharge, at the end of which the ovum bursts forth from its envelop.

"M. Raciborski reports two cases, which he was so happy as to observe, in support of these propositions. The first was that of a healthy, well-formed virgin, twenty-six years of age, who had had her regular menstrual discharge on the 30th August, 1842. On the 10th of September, seventeen days after, she was attacked with dysentery, which terminated fatally on the 30th of September, or one month after her last menses.

"Her hymen, after death, was found intact, allowing but a small passage. The right ovary, sensibly larger than the left, presented a mammillated vesicle, covering with its base part of the anterior face, and having upon its surface some vascular ramifications, while its internal membrane was found covered with a rich and delicate net-work of vessels. This membrane was yellowish-white, scarcely a quarter of a line thick, and easily separated from the other coats, and contained within it a liquid which, under the microscope, presented a large number of white granulations, with many yellowish globules. A small cicatrix, with a red areola, was found on the posterior surface of the ovary, corresponding with a small excavation, large enough to contain a cherry-stone, covered by a membrane evidently folded, and, as it were, festooned at its edges, of an orange-yellow tint, and containing at the bottom a small softish black clot of blood, attached, by some filaments only, to the sides of the excavation.—pp. 421-4.

"This last was unquestionably the trace of the previous maturation and separation of an ovum, and, without doubt, the Graafian vesicle, so much enlarged, would have been opened, had not the disease interfered with its entire changes.

"Another observation is given of a girl, nineteen years old, strongly formed, &c., who was attacked with scarlatina, having had her regular menstrual discharge twenty-four days previously. She was sent to La Charité, where, two days after being attacked, she died. On examination, the two ovaries were found of greatly different volume, the left being the larger; on this a large protuberance was observed, with a spot upon its most prominent part, of a deep red color, as large as a ten cent piece, surrounded with a broad areola of a lighter color, and gradually losing itself in that of the envelop of the ovary. The spot was evidently the result of a strong congestion with effusion of blood into the thickness of the tissues. A crucial incision through it opened a cavity large enough to hold a very large cherry, filled with a granular matter resembling, exactly, the liquid of a Graafian vesicle, hardened by alcohol, (it had been preserved in alcohol for several days before opening it.) only it was strongly colored red. The internal membrane was no longer as vascular as in the preceding case; but presented no inteival between it and the other. Besides this cavity, there was another, one half smaller, formed by a vesicle destined, probably, for the subsequent *ponte.* The left Fallopian tube was united to the ovary by old cellular adherences, its extremity being folded inwards, thus stopping up entirely its abdominal opening. The right ovary showed only some traces of ancient cicatrices, and some vesicles about the size of a pea—the right tube was obliterated at its middle.—pp. 425, 428.

M. R. presents these two facts, showing the condition of the vesicles at about the time of the menstrual discharge, but has never had occasion to observe any who have died during this period. Still, he concludes from analogy, and from some facts which are reported by others, that the rupture does not occur till the end of the menstrual evacuation. Upon examining a woman who had died from eight to twelve days after the cessation of her menstrual period, he says, you will always meet with traces of a rupture entirely too recent to be attributed to a later period than the last menses.—p. 432. One of the ovaries presents, then, a slightly prominent swelling, surmounted by a red spot, with a linear slit, of which the edges are already agglutinated together. Upon the ovary at this spot, a cavity will be observed, already smaller than that of the vesicle before the rupture, but filled entirely with a clot of blood, generally about the size of a moderate-sized cherry. M. R. has never, in forty cases, found this to fail. These clots may be easily removed, and the parietes of the vesicles generally present an orange-yellow color, which disappears in alcohol. The surface of the membrane is slightly folded, and, as it were, tomentous, the place where the opening had been being usually more translucent than the rest of the anterior surface. M. R. considers these clots, like the fleshy masses in the vesicles of animals, as a sort of mould to prevent the too rapid retraction of the parietes of the vesicle, a retraction which commences as soon as it is broken. The folds of the internal membrane which had formed immediately after the rupture of the vesicle, disappear in consequence of their adhesion; the more liquid portions of the clot are gradually absorbed, the clot itself becoming smaller, the cavity constantly contracting, until, at about the end of a month, it could hardly contain a cherry stone, as was observed in the first case above. Finally, the parietes are brought together, a single yellowish or slate-colored streak, or one < shaped, being the only trace left at about the end of from four to six months, generally. As long as the least trace of yellow color remains, there still exist some traces of the internal membrane, its yellow color being derived from an imbibition of the coloring matter of the blood.

"Death occurring after an acute disease to a woman, habitually regular every month, will reveal in her ovaries marks corresponding with those above mentioned in every stage of development, while, if the disease had been chronic, of very long standing, the functions of the ovaries, like those of other organs, being arrested, they will either exhibit old cicatrices, or the vesicles will be pale and without injection, even if large. M. Raciborski remarks, as has been done by others, that the notion that the ovaries act alternately is entirely incorrect. He says, also, that in cases where obstructions in the Fallopian tube exist, so that the ovum cannot pass through it, the ovum may fall into the cavity of the abdomen, and be dissolved without causing inflammation, the obstruction giving rise, as where the ovary is surrounded by false membranes, to physical sterility.

"Thus the same phenomena are observed in women that are ascertained to be present in the females of animals; and the same argument by analogy, offered by M. Pouchet, relative to this point, is maintained. The hæmorrhage from the uterus is merely an accessory to the phenomena of the periodical ripening of the ova, so that if animals do not present this, they do not therefore differ in the main and essential points connected with the function under consideration. Be this, however, as it may, many animals are known to present even this phenomenon, so that we must acknowledge the entire accordance in all essential matters between man and animals in relation to the function of generation.

"Before leaving this part of our subject, we will advert to the opinion of M. Raciborski, in relation to the nature of the menstrual hæmorrhage: 'It is nothing but a critical termination of the congestion which accompanies the highest degree of development of the Graafian vesicles. From all we have seen,' says he, 'it is probable that it coincides with the hæmorrhage which takes place in the interior of the vesicle some days before the *ponte*.'—p. 446. 'It cannot, therefore, differ in its nature from that of the blood, which constitutes the element of these congestions, and should be composed chiefly of arterial blood, as experience proves it to be.'—p. 447. The experiments of Denis and Bouchardat show that the fluid of the menstrual discharge differs from arterial blood only in being mixed with a certain proportion of mucus. But menstrual blood does not coagulate: M. Mandl has shown that the smallest quantity of mucus or pus mingled with blood prevents its coagulation; and it is known, that where large quantities are retained in the uterus, a symptom of dysmenorrhœa, clots are formed. We have thus fully exposed the opinions of M. Raciborski, which will be found as valuable, as they are admirably laid down by this most agreeable writer. To this statement of facts we find little to add from the work of M. Bischoff, whose observations relate rather to the inferior animals than to man. He states, however, that he has been so fortunate as to have had an opportunity of examining four healthy young women, of whom three were found dead in the water and the other died suddenly. In all, the evidences of menstruation at the time of their death were undoubted, and in three of them, at the same time, a Graafian vesicle in one of the ovaries was found burst and filled with fluid blood; and in the fourth, one uncommonly swollen was observed about seven lines in diameter. In one case, he afterwards learned that the person was actually menstruating at the time of her death. From Dr. Ecker, of Heidelberg, he has an account of a young person, aged 25, who was executed, having menstruated twelve days previously. Dr. E. found in one of her ovaries a Graafian vesicle, burst and filled with a fresh coagulum of blood. He was unable to find any thing of the ovum, as might be supposed at so distant a time.

"We have thus accumulated a mass of evidence, which might be greatly augmented if it were desirable, showing the correspondence of the rutting season or heat in animals with the menstrual period of women; and we may conclude this portion of our notice with another law of M. Bischoff, which he doubts not will hereafter be found true in all particulars:—'During the years in which a woman is susceptible of impregnation, an ovum ripens and is separated from the ovary every four weeks, this phenomenon being accompanied by simultaneous hæmorrhage from the uterus. This periodical maturation of an ovum is the first and most essential condition of conception and pregnancy. At this time alone will cöitus be followed by conception; at all others this last will be impossible.'—p. 43.

"It is almost unnecessary to remark, that during pregnancy, and for some time during lactation, this last period, varying in different women, there is a cessation of the function of secretion, if such a term be allowable, on the part of the ovaries. We have already adverted to the opinions of our authors, upon the length of time during which impregnation is possible at each menstrual period; and we have, also, noticed an objection which may be started against this theory, and which is satisfactorily answered by our authors, viz., that women often conceive who have never menstruated—an objection which is rather specious than real, for it has been shown, that menstruation or the bloody discharge is not essential to indicate the maturation of the ovum. It may ripen, as in many animals it does, and may be discharged, without the slightest appearance of hæmorrhage."

The Review, of which the above is an extract, may be found, over the signature C. R. K., in the *American Journal of the Medical Sciences* for January, 1845, p. 113–131.

